# Lysosomal-associated transmembrane protein 5 deficiency exacerbates cerebral ischemia/reperfusion injury

**DOI:** 10.3389/fnmol.2022.971361

**Published:** 2022-08-15

**Authors:** Zongyong Zhang, Lei Wang, Zhen Wang, Tingbao Zhang, Min Shi, Can Xin, Yichun Zou, Wei Wei, Xiang Li, Jincao Chen, Wenyuan Zhao

**Affiliations:** ^1^Department of Neurosurgery, Zhongnan Hospital of Wuhan University, Wuhan University, Wuhan, China; ^2^Department of Neurosurgery, Huanggang Central Hospital, Huanggang, China; ^3^Medical Research Institute, Wuhan University, Wuhan, China

**Keywords:** LAPTM5, lysosomal-associated protein transmembrane 5, stroke, ischemia- reperfusion injury, oxygen and glucose deprivation (OGD), ASK1 (apoptosis signal regulating kinase 1)

## Abstract

Lysosomal-associated transmembrane protein 5 (LAPTM5) has been demonstrated to be involved in regulating immunity, inflammation, cell death, and autophagy in the pathophysiological processes of many diseases. However, the function of LAPTM5 in cerebral ischemia-reperfusion (I/R) injury has not yet been reported. In this study, we found that LAPTM5 expression was dramatically decreased during cerebral I/R injury both *in vivo* and *in vitro*. LAPTM5 knockout (KO) mice were compared with a control, and they showed a larger infarct size and more serious neurological dysfunction after transient middle cerebral artery occlusion (tMCAO) treatment. In addition, inflammatory response and apoptosis were exacerbated in these processes. Furthermore, gain- and loss-of-function investigations in an *in vitro* model revealed that neuronal inflammation and apoptosis were aggravated by LAPTM5 knockdown but mitigated by its overexpression. Mechanistically, combined RNA sequencing and experimental verification showed that the apoptosis signal-regulating kinase 1 (ASK1)-c-Jun N-terminal kinase (JNK)/p38 pathway was mainly involved in the detrimental effects of LAPTM5 deficiency following I/R injury. Specifically, LAPTM5 directly interacts with ASK1, leading to decreased ASK1 N-terminal dimerization and the subsequent reduced activation of downstream JNK/p38 signaling. In conclusion, LAPTM5 was demonstrated to be a novel modulator in the pathophysiology of brain I/R injury, and targeting LAPTM5 may be feasible as a stroke treatment.

## Introduction

Stroke is one of the leading causes of disability and death globally (Campbell et al., 2019), resulting in approximately 5.5 million deaths annually, of which ischemic stroke accounts for 71% (GBD 2016 Lifetime Risk of Stroke Collaborators, Feigin et al., 2018; Lindsay et al., 2019). The treatment cycle for stroke patients is long, and the survivors typically cannot take care of themselves, resulting in a heavy social-economic burden.

Tissue plasminogen activator (tPA) is currently the only thrombolytic drug approved by the FDA for stroke treatment. However, the narrow therapeutic time window and complications after reperfusion result in the unsatisfactory prognosis of strokes. In addition, various complex pathophysiological processes are involved in cerebral ischemia-reperfusion (I/R) injury, including inflammation, apoptosis, oxidative and nitrative stress, and excitotoxicity (Chamorro et al., 2016; Nagy and Nardai, 2017; Zhang et al., 2021). Research on neuroprotective drugs for these potential targets has been robust, but the efficacy is mostly limited to animal experiments (Bosetti et al., 2017). Therefore, we need to further understand the pathological process of cerebral I/R injury and explore more effective treatment methods.

The lysosomal-associated transmembrane protein (LAPTM) family are multi-transmembrane proteins located on the lysosomal membrane, currently composed of LAPTM4 (LAPTM4a and LAPTM4b) and LAPTM5 (Maeda et al., 2005). LAPTM5 is a 30-kDa evolutionally conserved protein containing five predicted transmembrane domains (Adra et al., 1996). The N-terminus of LAPTM5 is located inside the lysosome, while the C-terminus is located in the cytoplasm (Ouchida et al., 2010). It is predominantly expressed in immune and hematopoietic cells (Scott et al., 1996) (Adra et al., 1996). However, LAPTM5 has been found to be dysregulated in many diseases. Origasa et al. (2001) found that LAPTM5 is upregulated in the retina after optic nerve injury, whereas LAPTM5 inactivation has been observed in human multiple myeloma (Hayami et al., 2003). Furthermore, Myles et al. (2007) showed that LAPTM5 is a susceptibility gene for inflammatory bowel disease, and it also plays an important role in the diagnosis and prognosis of testicular germ cell tumors (Li et al., 2021). Moreover, the LAPTM5 transcription level is often reduced in various cancer cell lines, which is significantly associated with poor prognosis (Nuylan et al., 2016). The methylation levels of LAPTM5 are decreased during normal lung development and are closely related to the differentiation status of lung tumors (Cortese et al., 2008). In addition, LAPTM5 acts upstream of various signaling pathways, participates in various physiological activities such as cell inflammation, death, and autophagy. Its deficiency can reduce the activation of NF-κB and MAPK signaling pathways, leading to decreased release of pro-inflammatory factors in macrophages (Glowacka et al., 2012). LAPTM5 overexpression in several cancer cells induces lysosomal cell death (Nuylan et al., 2016). In HeLa cells, LAPTM5 overexpression is involved in activating mitochondrial-dependent apoptosis pathways (Jun et al., 2017). Moreover, LAPTM5 can remarkably reduce autophagy activity (Hu et al., 2014). The regulation of these cellular physiological activities is highly correlated to the prognosis of cerebral I/R injury, and therefore, it is believed that LAPTM5 may play an unrecognized role during these processes.

In this study, LAPTM5 was observed to be downregulated during cerebral I/R injury both *in vivo* and *in vitro*. In addition, we demonstrated that LAPTM5 deficiency aggravated brain damage by exacerbating post-ischemic inflammation and apoptosis. However, the opposite results were observed in LAPTM5-overexpressing neurons. Mechanistically, the LAPTM5-regulated ASK1-JNK/p38 axis may play a crucial role in cerebral I/R injury. Thus, targeting LAPTM5 may be a promising therapeutic strategy for strokes.

## Materials and methods

### Animals

The animal experimental protocols were approved by the Animal Care and Use Committee of Zhongnan Hospital of Wuhan University. Animals received humanistic care in accordance with the National Institute of Health Guide for the Care and Use of Laboratory Animals. In this study, male mice aged 10–12 weeks (26–30 g) with a C57BL/6 background were used. For global LAPTM5 knockout mice construction, we designed a single-guide RNA (sgRNA; sequence: CCAGGGCTATGGTGGCGACT) targeting the upstream exon of LAPTM5; this was subsequently cloned into the pUC57-T7-sgRNA vector (51132; Addgene, Watertown, MA, United States). Cas9 mRNA and sgRNA were generated and co-injected into the single-cell fertilized eggs of C57BL/6 mice, and the injected fertilized eggs were then transplanted into the surrogate female mice. Thereafter, F0 generation mice were obtained after approximately 19–21 days of pregnancy. DNA was extracted from the ear tissue of mice aged 2 weeks and identified using check primers (F: 5′- GGGCCCAAGACTCCTTACTC-3′; R:5′- CCCAGACTCCCCAATACTCA -3′). Animals were housed under a comfortable temperature (22–24°C), humidity (40–70%), and light-controlled (12-h light/dark cycle) environment. All experimenters were blind to mouse genotype.

### Transient middle cerebral artery occlusion model in mice

Transient middle cerebral artery occlusion was performed in mice as previously described (Zhang et al., 2021). Briefly, mice are anesthetized by inhaling 2.0% isoflurane and oxygen/nitrous oxide gas mixture. First, the left common carotid artery was exposed, after which a silicon-coated monofilament (Cat# 602156PKRe; Doccol Corporation, Sharon, MA, United States) was inserted into the internal carotid artery via a cut in the external carotid artery until it occluded the base of the middle cerebral artery. Blood flow was restored 45 min later by withdrawing the monofilament and brain reperfusion lasted for 24 h. The monofilament was removed immediately after cerebral blood flow reduction in the sham operation control group. The mice’s anal temperature was maintained at 37 ± 0.2°C with a heating pad during operation. Doppler ultrasound was used to monitor the reduction and restoration of left cerebral blood flow. A successful tMCAO model was defined as CBF reduction by more than 75% in the ischemic phase, and then CBF was restored to at least 60% of the baseline after reperfusion. Mice were returned to a 37°C incubator for 2 h to recover. Water and food were freely available.

### Neurological function evaluation

After 24-h reperfusion, neurological function evaluation was conducted using a 9-point scale (Chen et al., 2014), as shown in Table 1. Afterward, the mice were anesthetized with 3% pentobarbital sodium (50 mg/kg, intraperitoneal injection) and perfused transcardially with saline followed by 4% paraformaldehyde, after which their brain tissue was quickly removed.

**TABLE 1:** Neurological function scale.

Neurological impairment symptoms	Score
No neurological deficits	0
The left forelimb is flexed, or the right forelimb cannot be fully extended when the tail is suspended	1
The left shoulder is adducted when the tail is suspended	2
Reduced resistance to a push to the left side	3
Moving spontaneously but turning to the left when dragged by the tail	4
Only spontaneously hovering or walking left	5
Moving only in response to a stimulus	6
No response to stimuli	7
Stroke-related death	8

### Infarct volume measurement

2,3,5-triphenyl-2H-tetrazolium chloride (TTC) staining was performed to calculate infarct volume after 24-h reperfusion following tMCAO. Briefly, mice brains were rapidly removed following deep anesthesia with 3% pentobarbital sodium (50 mg/kg, intraperitoneal injection) and frozen at –20°C for 30 min. Thereafter, brains were cut into seven consecutive 1-mm coronal slices. The sections were then immediately placed in 2% TTC and incubated at 37°C for 10 min. Subsequently, normal brain tissue was stained red, whereas the pale area was recognized as the infarct. Data were analyzed using Image-Pro Plus 6.0 software (Media Cybernetics Inc., Rockville, MD, United States). Edema volume (%) = (the volume of the ipsilateral hemisphere - volume of the contralateral hemisphere)/(contralateral volume × 2) × 100%. Infarct volume (%) = (Volume of the contralateral hemisphere - the volume of the non-lesioned ipsilateral hemisphere)/(contralateral volume × 2) × 100%.

### Immunofluorescence staining

Immunofluorescence staining was conducted as described previously (Zhang et al., 2021). Briefly, the cerebellum and olfactory bulb were excised from the removed brain. Subsequently, the brains were dehydrated in a gradient sucrose solution at 4°C (20% sucrose solution for 8 h followed by 30% sucrose solution overnight). After embedding the brains in opti-mum cutting temperature (OCT) compound (Cat# 4583; Sakura Finetek USA, Inc., Torrance, CA, United States), frozen sections were cut using a freezing microtome (Cat# CM1950; Leica Microsystems, Wetzlar, Germany).

For F4/80 staining, the sections were incubated with primary antibodies (anti-F4/80, Cat# MCA497; Bio-Rad AbD Serotec Limited, Oxford, United Kingdom) overnight at 4°C. Afterward, the sections were washed in phosphate buffered saline (PBS) thrice and incubated with secondary antibodies (anti-rat IgG (H+L); Cat# 4417; Cell Signaling Technology, Danvers, MA, United States) for 1 h at 37 °C. TUNEL staining was performed using a TUNEL staining kit (Cat# 11684817910; Roche Holding AG, Basel, Switzerland) according to the manufacturer’s instructions. The brain sections were incubated with primary antibodies (anti-NeuN; Cat# 26975-1-AP; Proteintech Group, Rosemont, IL, United States) overnight at 4°C. Goat anti-rabbit IgG (Cat# A11036; Invitrogen, Waltham, MA, United States) was applied as the secondary antibody. The nuclei were labeled with DAPI (Cat# 0100-20; SouthernBiotech, Birmingham, AL, United States).

### Immunohistochemistry

After the brain sections were incubated with EDTA (pH 9.0) and heated in a water bath, they were treated with H_2_O_2_ and blocked with 10% bovine serum albumin (NA8692; Bomei Biotechnology, Hefei, China). Subsequently, the sections were incubated at 4°C overnight with a primary antibody (anti-p-p65, 3033; Cell Signaling Technology). Next, the sections were incubated using a Rabbit Two-step Detection Kit (PV-9001; ZSGB-BIO, Beijing, China). Finally, the sections were visualized using diaminobenzidine (ZLI-9018; ZSGB-BIO) and hematoxylin was used to label the nuclei.

### Brain samples collection

For brain samples in western blot, real-time quantitative PCR (RT-qPCR), and RNA-seq experiments. The mice were anesthetized with 3% pentobarbital sodium (50 mg/kg, intraperitoneal injection) and perfused transcardially with saline, after which their brain tissue was quickly removed. The olfactory bulbs and front and back 1 mm of the brain tissue were excised. The ipsilateral (including the infarct and peri-infarct areas) and contralateral (or normal) hemispheres of the remaining brain tissues were harvested.

### Real-time quantitative PCR

Total RNA was extracted using TRIzol (Cat# T9424; Sigma Aldrich, St Louis, MO, United States). RNA was reverse-transcribed into cDNA using a Transcriptor First Strand cDNA Synthesis Kit (Cat# R323-01; Vazyme Biotech Co., Nanjing, China) according to the manufacturer’s protocol. RT-qPCR analysis was performed using a ChamQ SYBR Master Mix (Cat# Q311-03; Vazyme Biotech Co) and a LightCycler 480 QPCR System (Roche Holding AG). The results were normalized to the internal control β-actin. The primers used are shown in Table 2.

**TABLE 2:** Primer sequences for PCR.

Gene	Forward	Reverse
*Laptm5* (mouse)	AGTGGCCTTTATCACCGTGC	TGGCCGAATTCATGTGCTTC
*Laptm5* (rat)	AGCCCTGGCCATCTACCATA	CGGTTCTTGACCACTCCGAA
*Bcl2* (mouse)	CTTCTCTCGTCGCTACCGTC	CAATCCTCCCCCAGTTCACC
*Bax* (mouse)	TGAGCGAGTGTCTCCGGCGAAT	GCACTTTAGTGCACAGGGCCTTG
*Bax* (rat)	AGGACGCATCCACCAAGAAG	CAGTTGAAGTTGCCGTCTGC
*Bad (mouse)*	CCAGAGTTTGAGCCGAGTGAGCA	ATAGCCCCTGCGCCTCCATGAT
*Fas* (mouse)	CTGCGGAAACTTCAGGAAATG	GGTTCGGAATGCTATCCAGG
*Fas* (rat)	CCCGGACCCAGAATACCAAG	GTTCGTGTGCAAGGCTCAAG
*Tnf* (mouse)	CATCTTCTCAAAATTCGAGTGACAA	TGGGAGTAGACAAGGTACAACCC
*Tnf* (rat)	ATGGGCTCCCTCTCATCAGT	GCTTGGTGGTTTGCTACGAC
*Il6*(mouse)	TCCAGTTGCCTTCTTGGGAC	GACAGGTCTGTTGGGAGTGG
*Ccl2* (mouse)	TACAAGAGGATCACCAGCAGC	ACCTTAGGGCAGATGCAGTT
*Ccl2* (rat)	TGATCCCAATGAGTCGGCTG	GGTGCTGAAGTCCTTAGGGT
*Ccl5* (mouse)	TGCTGCTTTGCCTACCTCTC	TCTTCTCTGGGTTGGCACAC
*Ccl5* (rat)	CTGCTGCTTTGCCTACCTCT	TCTTCTCTGGGTTGGCACAC
*β-actin* (mouse)	GTGACGTTGACATCCGTAAAGA	GCCGGACTCATCGTACTCC
*β-actin* (rat)	CCGCGAGTACAACCTTCTTG	TGACCCATACCCACCATCAC

### Western blot

Western blot was performed as described previously (Zhang et al., 2021). Cells or tissues were lysed using radioimmunoprecipitation assay lysis buffer with protease (Cat# 04693132001; Roche Holding AG) and phosphatase inhibitor tablets (Cat# 4906837001; Roche Holding AG). A BCA protein quantification kit (Cat# 23225; Thermo Fisher Scientific, Waltham, MA, United States) was used to determine the protein concentration. Protein supernatants of the same quality were mixed with the loading buffer and boiled for 15 min at 95°C. Afterward, the protein samples were separated by 10% sodium dodecyl sulfate (SDS)-polyacrylamide gel electrophoresis (PAGE) and subsequently transferred to a PVDF membrane (Cat# IPVH00010; MilliporeSigma, Burlington, MA, United States). The membrane was incubated with primary antibodies overnight at 4°C. After washing in TBST thrice, the membrane was incubated with the corresponding secondary antibodies (1:10,000) at 23 ± 2°C for 1 h. Subsequently, the membranes were treated with ECL reagents (1705062; Bio-Rad Laboratories, Hercules, CA, United States) before visualization using a Bio-Rad imaging system (ChemiDoc™ XRS+). GAPDH served as an internal control. All antibodies used are listed in Table 3.

**TABLE 3:** Antibody information for western blot.

Antibody	Cat No.	Manufacturer
Laptm5	A17995	Abclonal
p-IKKβ	2694	CST
IKKβ	A0714	Abclonal
p-P65	3033	CST
P65	8242	CST
IκBα	4814	CST
Bcl2	3498	CST
Cleaved-Caspase3	9664	CST
Bax	2772	CST
p-ERK	4370	CST
ERK	4695	CST
p-JNK	4668	CST
JNK	9252	CST
p-p38	4511	CST
p38	8690	CST
p-ASK1	AF8096	Affinity
ASK1	A6274	Abclonal
Flag	M185-3L	MBL
HA	3724	CST
Myc	M047-3	MBL
GAPDH	2118	CST

### Hierarchical clustering analysis

The unweighted average distance (unweighted arithmetic mean group method, UPGMA) algorithm was employed for hierarchical cluster analysis to construct a phylogenetic tree of the samples. The HCLUST function of the R package was also used to better visualize the map of gene expression data.

### Differentially expressed genes analysis

The single-ended library was sequenced using an MGISEQ-2000 platform (MGI Tech Co., Ltd, Shenzhen, China), and the reading length was 50 bp. The sequenced fragments were compared to the mouse reference genome (mm10/GRCm38) using HISAT2 software, and the files obtained in the above steps were converted into binary BAM format using SAMtools. StringTie was used to calculate the fragment values per million genes for each exon model. Subsequently, DESeq2 identified differentially expressed genes (DEGs) based on the following two criteria: (1) a fold change of > 1.5 and (2) an adjusted *p*-value of < 0.05.

### Gene set enrichment analysis

The analysis was performed on the Java GSEA (version 3.0) platform with the “signal2noise” metric. Gene sets with *p-*values less than 0.05 and false discovery rate (FDR) less than 0.25 were considered statistically significant.

### Kyoto encyclopedia of genes and genomes pathway enrichment analysis

The biological pathway annotations for all genes were downloaded from the kyoto encyclopedia of genes and genomes (KEGG) database. KEGG pathway enrichment analysis was performed on all DEGs using Fisher’s exact test, and pathways with a *p-*value less than 0.05 were defined as significant.

### Oxygen-glucose deprivation/reoxygenation model

As previously described (Zhang et al., 2021), primary cortical neurons were prepared from the cerebral cortex of newborn Sprague-Dawley rats (within 1–2 days). Briefly, the dissected brain cortices were digested at 37°C using 0.125% trypsin (GIBCO, Grand Island, NY, United States) for 15 min. DMEM/F-12 medium (Cat# 11320033; GIBCO) containing DNase and 10% fetal bovine serum (FBS) was then applied to inactivate the trypsin. Subsequently, the cell suspension was filtered and centrifuged at 1,500 rpm for 8 min, and the precipitated cells were then resuspended using DMEM/F-12 medium containing 10% FBS and 1% penicillin-streptomycin liquid. Finally, the cells were seeded on plates pre-coated with poly-L-lysine (10 mg/mL; Sigma-Aldrich). After 3 h of incubation, the medium was replaced with Neurobasal-A medium (Cat# 10888022; GIBCO) supplemented with 2% B27 (Cat# 17504044; GIBCO), which was changed every 48 h. After culturing the neurons for 7 days, subsequent experiments were performed.

The OGD/R model was constructed to mimic cerebral I/R *in vitro* (Zhang et al., 2021). In brief, the primary neurons were incubated with glucose-free DMEM (Cat# 11966025; GIBCO) under hypoxic conditions (95% N_2_ and 5% CO_2_) for 1 h followed by normal culturing for 24 h.

### Recombinant adenoviral vectors and neurons infection

LAPTM5-overexpressing adenovirus (Ad*LAPTM5*) was purchased from Hanheng Biology (Shanghai, China). To construct the LAPTM5 knockdown adenovirus, hairpin-forming oligonucleotides (Primers in Table 4) were synthesized, annealed, and cloned into pENTR-U6-CMV-GFP shuttle vector. Adenoviruses were generated using an AdEasy Adenoviral Vector System Kit (Cat# 240009; Agilent Technologies). The above plasmids were recombined with the pAdEasy backbone vector and then transfected into HEK293A cells using TurboFect transfection reagent (Cat# R0531; ThermoFisher Scientific). Recombinant adenoviruses were plaque-purified using cesium chloride density gradient centrifugation and verified by restriction digestion.

**TABLE 4:** Primer of three rat sh*LAPTM5* plasmid.

Primer	Sequence
F1	CCGG CGGTAAAGTGTCCTGTAGGTT CTCGAG AACCTACAGGACACTTTACCG TTTTTG
R1	AATTCAAAAA CGGTAAAGTGTCCTGTAGGTT CTCGAG AACCTACAGGACACTTTACCG
F2	CCGG GCTAGACTTCTGTTTGAGTAT CTCGAG ATACTCAAACAGAAGTCTAGC TTTTTG
R2	AATTCAAAAA GCTAGACTTCTGTTTGAGTAT CTCGAG ATACTCAAACAGAAGTCTAGC
F3	CCGG GCACAGCCAGTTCATCAACAT CTCGAG ATGTTGATGAACTGGCTGTGC TTTTTG
R3	AATTCAAAAA GCACAGCCAGTTCATCAACAT CTCGAG ATGTTGATGAACTGGCTGTGC

For the *in vitro* experiments, cultured neurons were transfected with adenoviruses at a multiplicity of infection (MOI) of 100 for 48 h before OGD/R treatment.

### Cell viability assay

The cell viability of the primary cultured neurons was assessed using a cell counting kit-8 (CCK-8) assay (Cat# 44786; Dojindo Molecular Technologies, Inc., Rockville, MD, United States) in accordance with the manufacturer’s protocol. After suffering from OGD/R, the neurons were incubated with CCK-8 reaction solution at 37°C for 2 h, and the number of viable cells was quantified by measuring the absorbance at 450 nm.

### Plasmid construct and transfection

Full-length LAPTM5 and ASK1 fragments and truncated ASK1 (1-678, 679-1374) were obtained through PCR from human cDNA and then subcloned into pcDNA5-Flag, pcDNA5-HA, pcDNA5-GST-HA, pcDNA5-myc vector. The constructed pcDNA5-Flag-LAPTM5 vectors were used as a template to amplify Flag-Laptm5 mutants [Flag-Laptm5(mUIM), Flag-Laptm5(mPY1), Flag-Laptm5(mPY2), Flag-Laptm5(mPY3), Flag-Laptm5(mPY1-3)]. The primers used for plasmid construction are shown in Table 5. All plasmid sequences were confirmed by gene sequencing.

**TABLE 5:** Primer sequences for plasmid construct.

Gene	Sequence (human)	
Flag-Laptm5	F	TCGGGTTTAAACGGATCCATGGACCCCCGCTTGTCCACTG
	R	GGGCCCTCTAGACTCGAGTCACACCTCTGAGTATGGGGGT
HA-ASK1	F	TCGGGTTTAAACGGATCCATGAGCACGGAGGCGGACG
	R	GGGCCCTCTAGACTCGAGTCAAGTCTGTTTGTTTCGAAAG
GST-HA-ASK1	F	TCGGGTTTAAACGGATCCATGAGCACGGAGGCGGACG
	R	GGGCCCTCTAGACTCGAGTCAAGTCTGTTTGTTTCGAAAG
Flag-ASK1	F	TCGGGTTTAAACGGATCCATGAGCACGGAGGCGGACG
	R	GGGCCCTCTAGACTCGAGTCAAGTCTGTTTGTTTCGAAAG
GST-HA-Laptm5	F	TCGGGTTTAAACGGATCCATGGACCCCCGCTTGTCCACTG
	R	GGGCCCTCTAGACTCGAGTCACACCTCTGAGTATGGGGGT
HA-ASK1 (1-678)	F	TCGGGTTTAAACGGATCCATGAGCACGGAGGCGGACG
	R	GGGCCCTCTAGACTCGAGTCAATCATATTCATAGTCATACTCCAG
HA-ASK1(679-1374)	F	TCGGGTTTAAACGGATCCGAAAATGGTGACAGAGTCGTTTTAG
	R	GGGCCCTCTAGACTCGAGTCAAGTCTGTTTGTTTCGAAAG
Flag-Laptm5(mUIM)	F	TCGCGAAGGTGGGCGCGCCGTCCTACGAGGAAGC
	R	GCGCGCCCACCTTCGCGAGCATCTTGGAGTTTCTCTTC
Flag-Laptm5(mPY1)	F	TGCCCGCCGCCCTCAAGTTGGCCT
	R	CCAACTTGAGGGCGGCGGGCAGCTCAATGTA
Flag-Laptm5(mPY2)	F	TGCCGTCCGCCGAGGAAGCCCTGTCTTTG
	R	CTTCCTCGGCGGACGGCAGGACCACCT
Flag-Laptm5(mPY3)	F	TCGGGTTTAAACGGATCCATGGACCCCCGCTTGTCCACTG
	R	GGGCCCTCTAGACTCGAGTCACACCTCTGAGGCTGGGGGTGG
Myc-Laptm5	F	TCGGGTTTAAACGGATCCATGGACCCCCGCTTGTCCACTG
	R	GGGCCCTCTAGACTCGAGTCACACCTCTGAGTATGGGGGT

The 293T cells were transfected with the indicated plasmids and the medium was refreshed 6 h after transfection. Cells were harvested after culturing for 24 h.

### Immunoprecipitation

The collected HEK293T cells were lysed in ice-cold immunoprecipitation (IP) buffer for 30 min. After the cell lysate was centrifuged, 10% of supernatants were used as input, and the remainder was incubated with protein A/G-Agarose beads [AA104307; Bestchrom (Shanghai) Bioscience Co., Ltd., Shanghai, China] and the corresponding IP antibodies for 6 h at 4°C. The beads were then washed with high-concentration IP buffer twice and low-concentration IP buffer twice. Next, the binding protein was eluted by boiling at 95°C for 15 min with SDS loading buffer. Finally, western blot was conducted.

### Glutathione-S-transferase pull-down assay

The collected HEK293T cells were lysed in ice-cold IP buffer for 30 min. After the cell lysate was centrifuged, 10% of supernatant was used as input. The supernatant from HEK293T cells transfected with pcDNA5-GST-HA-*ASK1* (or *LAPTM5*) and its control pcDNA5-GST-HA was incubated with glutathione-Sepharose beads [AA0072; Bestchrom (Shanghai) Co., Ltd.] for 1 h. Additionally, the supernatant from HEK293T cells transfected with pcDNA5-Flag-*LAPTM5* (or *ASK1*) was then added to protein binding Glutathione-S-transferase (GST)-beads followed by incubation at 4°C for 6 h. Subsequently, the beads were washed with low-concentration IP buffer thrice. Next, the binding protein was eluted by boiling at 95°C for 15 min with SDS loading buffer. Finally, western blot was conducted.

### Statistical analysis

All data were expressed as the mean ± standard deviation (SD) and analyzed using SPSS 21.0 statistical software (SPSS Inc., Chicago, IL, United States). Shapiro–Wilk’s test was used to determine data normality. For normally distributed data, differences between two groups were compared using an unpaired Student’s *t*-test. Non-normally distributed data were compared using Mann–Whitney test. Statistical significance was considered at *p* < 0.05.

## Results

### LAPTM5 expression is downregulated after cerebral ischemia-reperfusion injury

**FIGURE 1:**
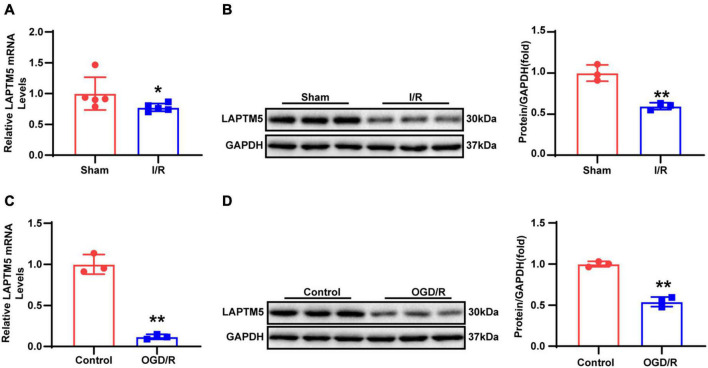
LAPTM5 expression is downregulated after cerebral I/R injury. **(A)** The LAPTM5 mRNA levels were downregulated after 24-h reperfusion following tMCAO, *n* = 5 mice per group, *p < 0.05 vs. sham. **(B)** Western blot showed that LAPTM5 decreased after 24-h reperfusion following tMCAO, *n* = 3 mice per group, **p < 0.01 vs. sham. **(C)** The mRNA levels of LAPTM5 were decreased in primary neurons treated with OGD for 1 h followed by reoxygenation for 24 h (OGD/R), *n* = 3 independent experiments, **p < 0.01 vs. control. **(D)** Western blot showed lower LAPTM5 expression in primary neurons treated with OGD/R, *n* = 3 independent experiments, **p < 0.01 vs. control.

To explore whether LAPTM5 plays a role in cerebral I/R injury, we first established an experimental murine model induced via tMCAO for 45 min followed by reperfusion for 24 h. Thereafter, the LAPTM5 mRNA and protein levels were tested using RT-qPCR and western blot, respectively. The results showed that LAPTM5 expression was decreased following tMCAO/reperfusion (Figures 1A,B). Furthermore, we isolated primary neurons and generated a stroke model *in vitro* induced by OGD/R. Both the mRNA and protein levels of LAPTM5 were reduced in primary neurons following OGD/R (Figures 1C,D). Overall, these findings suggested that LAPTM5 might be involved in cerebral I/R injury.

### LAPTM5 ablation aggravates brain ischemia-reperfusion injury

**FIGURE 2:**
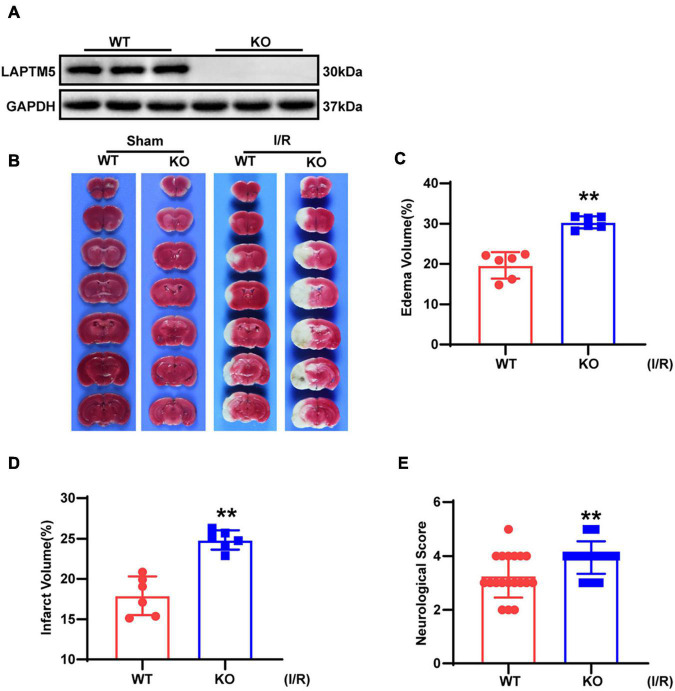
LAPTM5 ablation aggravates cerebral I/R injury. **(A)** Western blot of LAPTM5 expression in brains from WT and LAPTM5-KO (knockout) mice, *n* = 3 mice per group. **(B)** Representative images of brain TTC staining, *n* = 6 mice per group. **(C–E)** LAPTM5-KO mice showed larger edema **(C)** (*n* = 6 mice per group, **p < 0.01, vs. WT I/R) and infarct volumes **(D)** (*n* = 6 mice per group, **p < 0.01 vs. WT I/R) and more severe neurological deficits based on function evaluation **(E)** (*n* = 19–20 mice per group, *p < 0.05 vs. WT I/R) following I/R.

To elucidate the function of LAPTM5 in brain I/R injury, LAPTM5 KO mice were challenged with tMCAO/reperfusion treatment in parallel with WT mice. LAPTM5 KO mice were confirmed, as shown in Figure 2A. TTC staining was performed to determine the infarct area (Figure 2B). We found that LAPTM5 KO mice showed larger brain edema and infarct volume than the WT group after tMCAO/reperfusion (Figures 2C,D). Compared with the WT group, LAPTM5 KO mice exhibited more severe dysfunction based on neurological function evaluation (Figure 2E). Collectively, these results indicated that LAPTM5 ablation aggravates brain I/R injury.

### LAPTM5 deletion facilitates inflammation induced by cerebral ischemia-reperfusion injury

**FIGURE 3:**
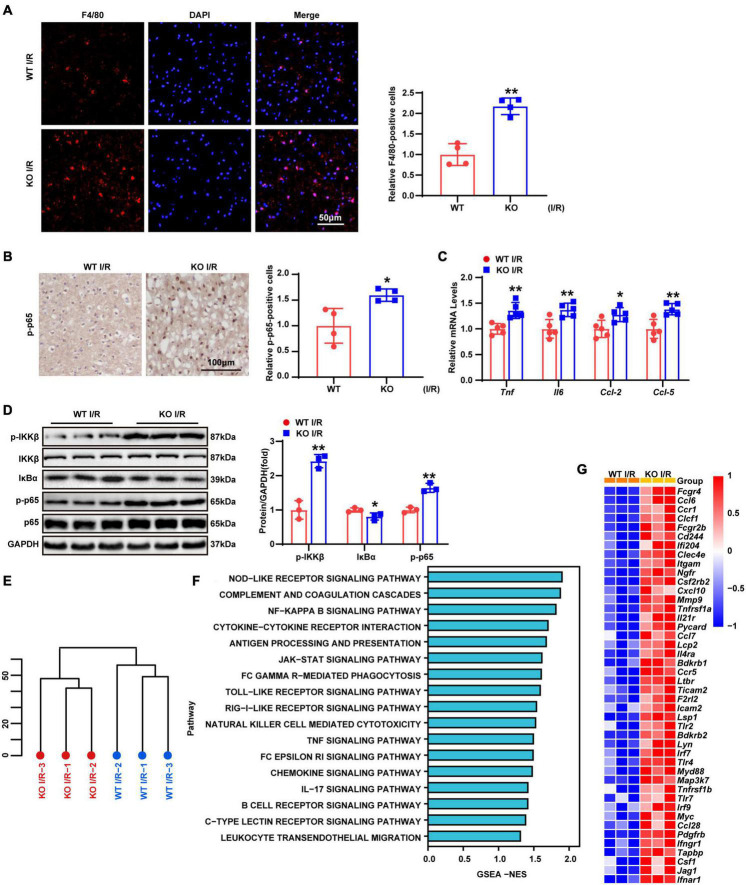
LAPTM5 deletion facilitates inflammation induced by cerebral I/R injury. **(A)** Representative images of brain F4/80 staining and quantification (right) showed more macrophages/microglia infiltrating the brains of LAPTM5 KO mice after I/R injury, *n* = 4 mice per group, **p < 0.01 vs. WT I/R. Scale bar, 50 μm. F4/80 (red), DAPI (blue). **(B)** Representative images of brain p-p65 staining and quantification (right) showed higher percentages of p-p65 positive cells in LAPTM5 KO group subjected to I/R injury, *n* = 4 mice per group, *p < 0.05 vs. WT I/R. Scale bar, 100 μm. **(C)** LAPTM5 KO upregulated the mRNA levels of proinflammatory genes (*Tnf, Il6, Ccl-2, and Ccl-5*) following I/R injury, *n* = 5 mice per group, *p < 0.05, **p < 0.01 vs. WT I/R. **(D)** Western blot revealed that the protein levels of phosphorylated IKKβ and p65 were increased, while that of IκBa was decreased in LAPTM5-KO mice after I/R 24 h, *n* = 3 mice per group, *p < 0.05, **p < 0.01 vs. WT I/R. **(E)** Hierarchical clustering analysis showed the global distribution profiles of sequencing data set from brains in WT and TBC1D25-KO mice (*n* = 3 mice per group). **(F)** Gene set enrichment analysis (GSEA) showed the inflammation-related pathway in LAPTM5-KO mice brains based on the RNA-seq data set (*n* = 3 mice per group). **(G)** Heatmap showing the differentially expressed inflammatory genes between LAPTM5-KO and WT mice brains (*n* = 3 mice per group).

Previous studies demonstrated that post-ischemic inflammation becomes a critical step in the pathophysiology of cerebral I/R injury (Anrather and Iadecola, 2016). Accordingly, F4/80 staining was performed to determine macrophages/microglia infiltrating the brain tissue. The results revealed that F4/80 positive cell numbers were significantly higher in LAPTM5 KO mice than those in the WT group (Figure 3A). In addition, immunohistochemical staining of p-p65 showed NF-κB activation. Compared with the WT group, the proportion of p-p65 positive cells in the KO group was significantly increased following brain I/R injury (Figure 3B). RT-qPCR indicated the elevated transcription levels of inflammatory genes (*Tnf, Il6, Ccl-2*, and *Ccl-5*) in the KO group (Figure 3C). Similarly, we tested the protein levels of NF-κB pathway-related molecules. In KO mice, the phosphorylation levels of IKKβ and p65 increased, while the expression levels of IκBα decreased after tMCAO/reperfusion treatment. The total IKKβ and p65 expression levels did not change significantly between the two groups (Figure 3D). Additionally, we performed RNA-seq in the brains of WT and LAPTM5-KO mice. Hierarchical clustering analysis showed the comparable distribution profiles of the samples (Figure 3E). Gene set enrichment analysis (GSEA) indicated that the inflammation-related pathways considerably differed between the two groups (Figure 3F). Similarly, LAPTM5 KO mice showed higher expression levels of inflammatory gene profiles, as shown by Heatmap (Figure 3G). Together, these data suggest that LAPTM5 deletion results in the deterioration of the inflammatory response in the process of cerebral I/R injury.

### LAPTM5 deletion promotes neuronal apoptosis induced by cerebral ischemia-reperfusion injury

**FIGURE 4:**
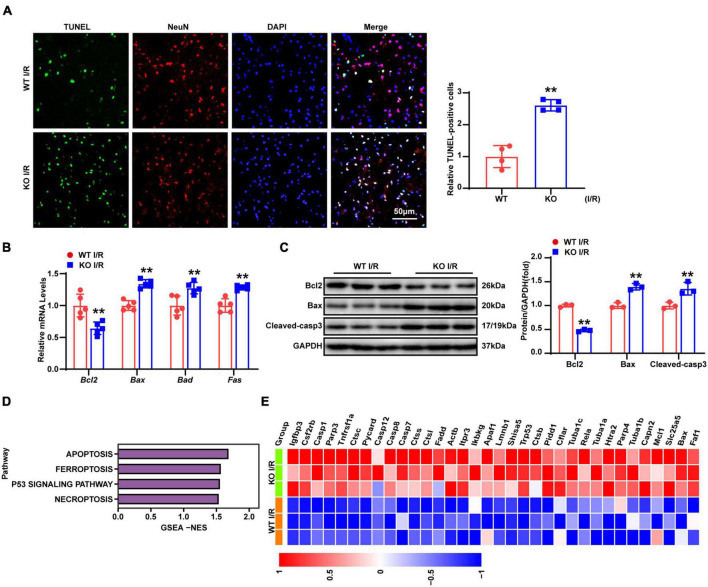
LAPTM5 deletion promotes neuronal apoptosis induced by cerebral I/R injury. **(A)** Representative images of brain co-stained for TUNEL (green), NeuN (red), and DAPI (blue), and the right panels (quantification) showed more apoptotic neurons in the LAPTM5-KO group after I/R. *n* = 4 mice per group, **p < 0.01 vs. WT I/R. Scale bar, 50 μm. **(B)** RT-qPCR showed decreased expression of anti-apoptotic genes (*Bcl2*), while pro-apoptotic genes (*Bax, Bad, and Fas*) expression were increased in the LAPTM5-KO group after I/R, *n* = 5 mice per group, **p < 0.01 vs. WT I/R. **(C)** Western blot showed decreased expression of anti-apoptotic proteins (Bcl2), while pro-apoptotic proteins (Bax and Cleaved-caspase 3) were increased in LAPTM5-KO mice after I/R injury. *n* = 3 mice per group, **p < 0.01 vs. WT I/R. **(D)** GSEA showed the expression levels of cell death-related pathways induced by LAPTM5 deficiency (*n* = 3 mice per group). **(E)** Heatmap showing cell death-related genes expression induced by LAPTM5 deficiency (*n* = 3 mice per group).

Neuronal apoptosis was demonstrated to contribute to stroke-related brain injury in many studies (Chen et al., 2020; Lai et al., 2020; Naito et al., 2020). TUNEL and NeuN co-staining were used to determine neuronal apoptosis. As shown by Figure 4A, there were considerably more TUNEL positive neurons in the KO group compared with those in the WT group after 24-h reperfusion following tMCAO. Furthermore, RT-qPCR showed decreased expression of the anti-apoptotic gene *Bcl2*, while pro-apoptotic gene (*Bax*, *Bad*, *and Fas*) levels were increased (Figure 4B). Similarly, the protein levels of Bcl2 were downregulated, while those of pro-apoptotic molecules (Bax and cleaved caspase-3) were upregulated (Figure 4C). To depict the overall molecular profile of apoptosis, we analyzed the RNA-seq data, and results revealed that apoptotic processes related to molecular events were more involved in the brains of TBC1D25-KO mice, as shown by Figures 4D,E. Overall, these results indicated that LAPTM5 deficiency exacerbates neuronal apoptosis during cerebral I/R injury.

### LAPTM5 knockdown aggravates neuronal inflammation and apoptosis in primary neurons treated with oxygen and glucose deprivation/reoxygenation

**FIGURE 5:**
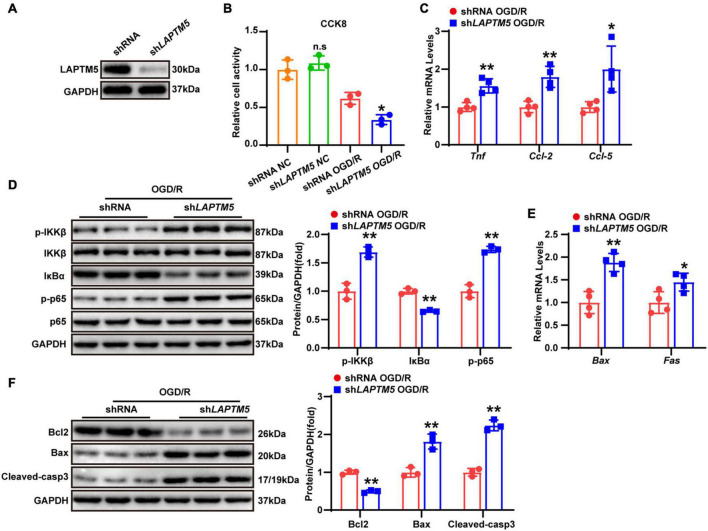
LAPTM5 knockdown aggravates neuronal inflammation and apoptosis in primary neurons treated with OGD/R. **(A)** Western blot of LAPTM5 expression in the control and LAPTM5 knockdown primary neurons. **(B)** CCK8 assays revealed lower cell viability in the LAPTM5 knockdown group after OGD/R treatment, while LAPTM5 knockdown did not affect viability of neurons cultured under normal conditions, *n* = 3 independent experiments, *p < 0.05 vs. the shRNA OGD/R group. n.s. indicates no significance vs. the shRNA NC group. **(C)** LAPTM5 knockdown upregulated the mRNA levels of proinflammatory genes (*Tnf, Ccl-2, and Ccl-5*) after OGD/R, *n* = 4 independent experiments, *p < 0.05, **p < 0.01 vs. the shRNA OGD/R group. **(D)** Western blot analysis showed that the protein levels of phosphorylated IKKβ and p65 were increased, while that of IκBα was decreased in the sh*LAPTM5* group after OGD/R, *n* = 3 independent experiments, **p < 0.01 vs. the shRNA OGD/R group. **(E)** RT-qPCR showed increased pro-apoptotic gene (*Bax, Fas*) expression in the sh*LAPTM5* group after OGD/R, *n* = 4 independent experiments, *p < 0.05, **p < 0.01 vs. the shRNA OGD/R group. **(F)** Western blot showed decreased expression of anti-apoptotic proteins (Bcl2), while pro-apoptotic proteins (Bax and Cleaved-caspase 3) were increased in the sh*LAPTM5* group after OGD/R, *n* = 3 independent experiments, **p < 0.01 vs. the shRNA OGD/R group.

As evidenced by previous research, several types of cells (including neurons, astrocytes, and endothelial and immune cells) in the brain tissue are involved in the pathophysiological process of strokes (Cheon et al., 2018a). However, global LAPTM5 KO mice were employed in the present study. Thus, we explored the role of LAPTM5 in OGD/R-treated primary neurons. Cells were infected with Adsh*LAPTM5* or AdshRNA before being subjected to OGD/R. LAPTM5 knockdown neurons were identified by western blot (Figure 5A), and cell viability was assessed using CCK8 assays. The results showed decreased viability in primary neurons after OGD/R treatment in the Adsh*LAPTM5* group, while cell viability of neurons cultured under normal conditions was not affected by LAPTM5 knockdown (Figure 5B). Compared with the control group, the transcriptional levels of pro-inflammatory genes (*Tnf, Ccl-2*, and *Ccl-5*) were dramatically increased in the Adsh*LAPTM5* group following OGD/R (Figure 5C). Determination of NF-κB pathway-related molecules via western blot revealed that the protein levels of p-IKKβ and p-p65 were increased, while those of IκBα were decreased in LAPTM5 knockdown primary neurons. Total IKKβ and p65 were not differentially expressed between the two groups (Figure 5D). In addition, LAPTM5 knockdown upregulated the mRNA levels of pro-apoptotic genes (*Bax* and *Fas*; Figure 5E). Moreover, western blot exhibited increased protein levels of pro-apoptotic molecules (Bax and cleaved caspase-3) and reduced expression levels of anti-apoptotic molecules (Bcl2; Figure 5F). Thus, in accordance with the *in vivo* results, LAPTM5 knockdown exacerbates neuronal inflammation and apoptosis in primary neurons treated with OGD/R.

### LAPTM5 overexpression attenuates neuronal inflammation and apoptosis in primary neurons following oxygen and glucose deprivation/reoxygenation

**FIGURE 6:**
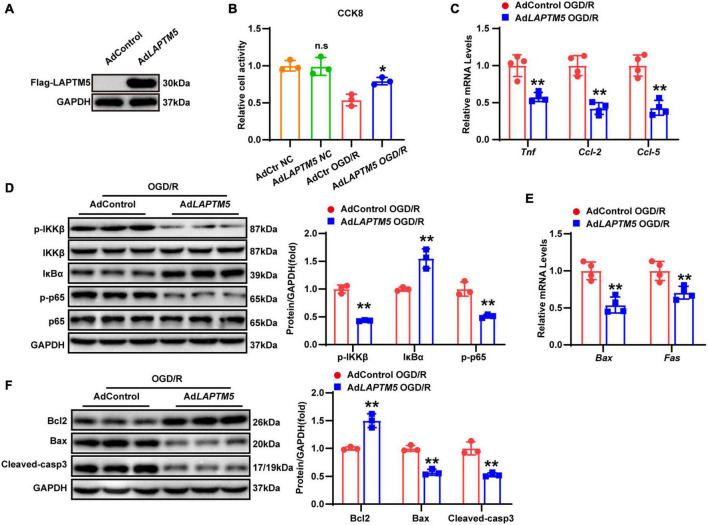
LAPTM5 overexpression attenuates neuronal inflammation and apoptosis in primary neurons following OGD/R. **(A)** Western blot of LAPTM5 expression in the control and LAPTM5 overexpressing primary neurons. **(B)** The LAPTM5 overexpressing group showed higher cell viability after OGD/R, while it did not affect viability of neurons cultured under normal conditions, *n* = 3 independent experiments, *p < 0.05 vs. the AdControl OGD/R group, n.s. indicates no significance vs. the AdControl NC group. **(C)** LAPTM5 overexpression decreased the mRNA levels of proinflammatory genes (*Tnf, Ccl-2, and Ccl-5*) after OGD/R, *n* = 4 independent experiments, **p < 0.01 vs. the AdControl OGD/R group. **(D)** Western blot analysis revealed that the protein levels of phosphorylated IKKβ and p65 were decreased, while that of IκBα was upregulated in the Ad*LAPTM5* group after OGD/R, *n* = 3 independent experiments, **p < 0.01 vs. the AdControl OGD/R group. **(E)** Primary neurons in the Ad*LAPTM5* group showed decreased pro-apoptotic gene (*Bax, Fas*) expression after OGD/R, *n* = 4 independent experiments, **p < 0.01 vs. the AdControl OGD/R group. **(F)** Western blot reflected higher expression of anti-apoptotic proteins (Bcl2), while pro-apoptotic proteins (Bax and Cleaved-caspase 3) were decreased in the Ad*LAPTM5* group after OGD/R, *n* = 3 independent experiments, **p < 0.01 vs. the AdControl OGD/R group.

To investigate the function of LAPTM5 overexpression in neurons, primary cultured neurons were infected with LAPTM5 overexpression adenovirus (Ad*LAPTM5*) or its control Ad*GFP* and then treated with OGD/R. LAPTM5 overexpressing neurons were confirmed by western blot (Figure 6A). CCK8 assays indicated that LAPTM5 overexpression increased neuronal viability following OGD/R treatment (Figure 6B). Furthermore, LAPTM5 overexpression downregulated the transcriptional levels of pro-inflammatory genes (*Tnf, Ccl-2, and Ccl-5*), as shown by the RT-qPCR results (Figure 6C). The protein levels of p-IKKβ and p-p65 were decreased, while those of IκBα were increased, as shown by western blot in the Ad*LAPTM5* group following OGD/R (Figure 6D). Meanwhile, LAPTM5 overexpression diminished the pro-apoptotic molecules (Bax, Cleaved-caspase3, and Fas) expression levels but elevated anti-apoptotic factor (Bcl2) expression (Figures 6E,F). In summary, LAPTM5 overexpression could ameliorate inflammation and apoptosis in primary neurons subjected to OGD/R treatment.

### LAPTM5 suppresses the ASK1-JNK/p38 signaling pathway during cerebral ischemia-reperfusion injury both *in vivo* and *in vitro*

**FIGURE 7:**
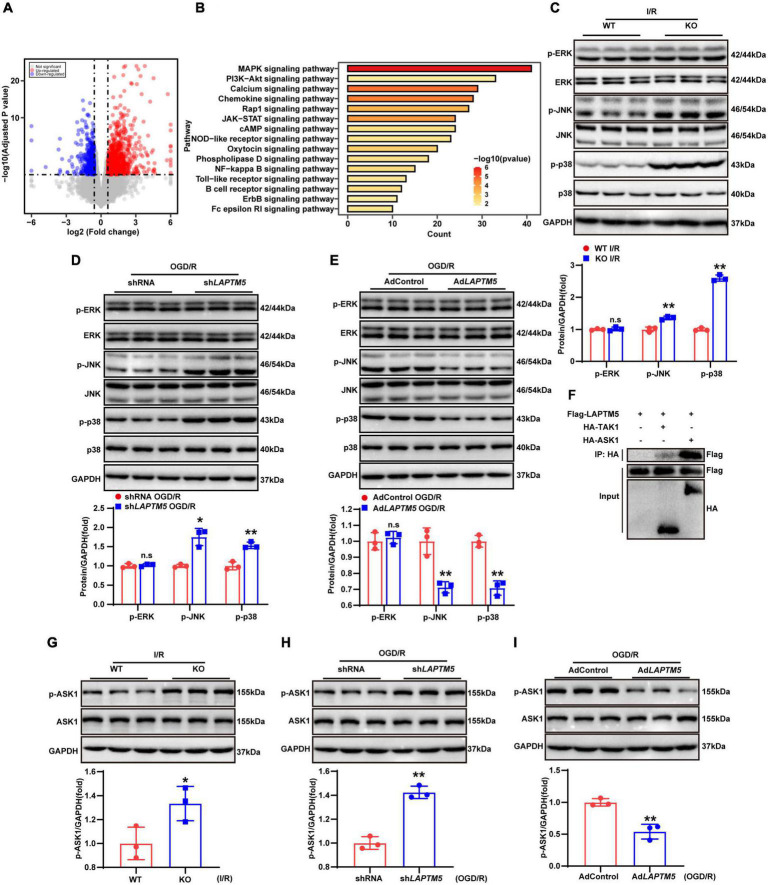
LAPTM5 suppresses the ASK1-JNK/p38 signaling pathway during cerebral I/R injury both *in vivo* and *in vitro*. **(A)** Volcano plots showing differentially expressed genes (DEGs) in WT and LAPTM5-KO mice brains, *n* = 3 mice per group. **(B)** The top 15 most significantly enriched pathways between WT and LAPTM5-KO groups are shown based on the Kyoto Encyclopedia of Genes and Genomes (KEGG) enrichment analysis of DEGs, *n* = 3 mice per group. **(C)** Western blot showed increased protein levels of phosphorylated c-Jun N-terminal kinase (JNK) and p38, while there was no difference in phosphorylated extracellular signal-regulated kinase (p-ERK) expression in LAPTM5-KO mice after I/R. *n* = 3 mice per group, **p < 0.01 vs. the WT I/R group, n.s. indicates no significance vs. the WT I/R group. **(D)** Western blot showed increased protein levels of phosphorylated JNK and p38, while there was no difference in p-ERK expression in the sh*LAPTM5* group after OGD/R, *n* = 3 independent experiments, *p < 0.05, **p < 0.01 vs. the shRNA OGD/R group, n.s. indicates no significance vs. the shRNA OGD/R group. **(E)** Western blot showed decreased protein levels of phosphorylated JNK and p38, while there was no difference in p-ERK expression in the Ad*LAPTM5* after OGD/R. *n* = 3 independent experiments, **p < 0.01 vs. AdControl OGD/R group, n.s. indicates no significance vs. AdControl OGD/R group. **(F)** Co-immunoprecipitation (co-IP) assays demonstrated that LAPTM5 could bind to ASK1, while it almost could not bind to TAK1 in 293T cells transfected with the indicated plasmids, *n* = 3 independent experiments. **(G)** Western blot showed increased protein levels of phosphorylated ASK1 (p-ASK1), while there was no difference in total ASK1 expression in LAPTM5-KO mice after I/R. *n* = 3 mice per group, *p < 0.05 vs. WT I/R group. **(H)** Western blot showed increased expression of p-ASK1, while no difference was observed in total ASK1 expression in the sh*LAPTM5* group after OGD/R, *n* = 3 independent experiments, **p < 0.01 vs. the shRNA OGD/R group. **(I)** Western blot showed decreased expression of p-ASK1, while no difference in total ASK1 expression was observed in the Ad*LAPTM5* after OGD/R. *n* = 3 independent experiments, **p < 0.01 vs. AdControl OGD/R group.

To investigate the underlying molecular mechanisms of LAPTM5 in cerebral I/R injury, we analyzed the RNA-seq data. DEGs and KEGG pathways enrichment analysis showed that the MAPK signaling pathway was predominantly involved in these processes (Figures 7A,B). Previous studies have also confirmed that the MAPK signaling pathway plays an important role in cerebral I/R injury (Lu et al., 2013; Sun and Nan, 2016). Therefore, we tested the total and phosphorylated levels of ERK, JNK, and p38. The results showed that LAPTM5 deficiency increased the phosphorylation levels of JNK and p38, while the total ERK, JNK, and p38 were not differentially expressed after cerebral I/R injury both *in vivo* and *in vitro*. Phosphorylation levels of ERK (p-ERK) were also not changed (Figures 7C,D). Conversely, the phosphorylation levels of JNK and p38 were decreased, while there were no differences in ERK, p-ERK, JNK, and p38 expression in LAPTM5 overexpressing primary neurons (Figure 7E). ASK1 and TAK1 are members of the mitogen-activated protein kinase kinase kinase (MAP3K) family. As upstream regulatory molecules of the JNK/p38 signaling pathway, they are involved in cerebral I/R injury (Kim et al., 2011; Zeyen et al., 2020). In 293T cells, immunoprecipitation (IP) experiments demonstrated that Flag-LAPTM5 could interact with HA-tagged ASK1, but it nearly could not bind to HA-tagged TAK1 (Figure 7F). Moreover, western blot analysis showed that LAPTM5 deficiency increased the phosphorylation levels of ASK1 both *in vivo* and *in vitro*, while there was no difference in total ASK1 levels (Figures 7G,H). Conversely, the phosphorylation levels of ASK1 were distinctly suppressed in LAPTM5 overexpressing primary neurons challenged with OGD/R (Figure 7I). Based on these findings, we conclude that LAPTM5 inhibits the ASK1-JNK/p38 signaling pathway during cerebral I/R injury both *in vivo* and *in vitro*.

### LAPTM5 inhibits ASK1 N-terminal dimerization by directly interacting with ASK1

**FIGURE 8:**
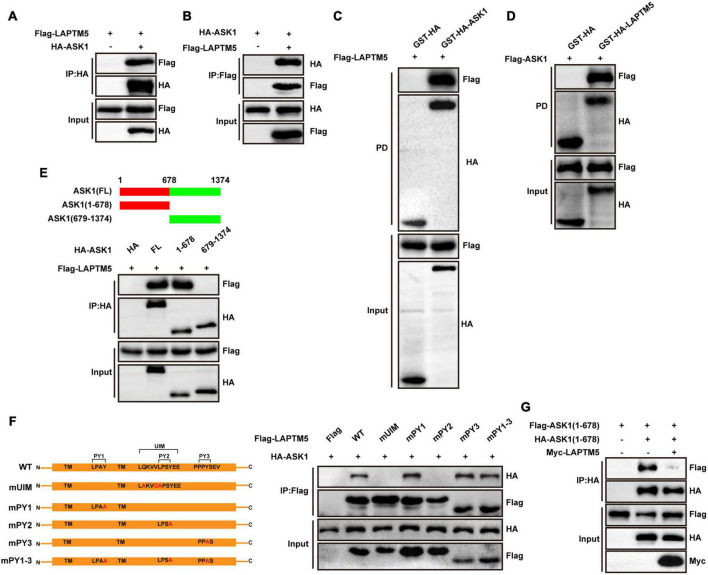
LAPTM5 inhibits ASK1 N-terminal dimerization by directly interacting with ASK1. **(A,B)** Co-IP assays were performed to show the interaction between LAPTM5 and ASK1 in 293T cells transfected with the indicated plasmids. **(C,D)** GST pull-down assays showed the direct interaction of LAPTM5 and ASK1 in 293T cells transfected with the indicated plasmids. **(E)** Top, schematic of full-length or truncated ASK1; bottom, Co-IP assay results revealed that the N-terminus of ASK1 [ASK1(1-678 aa)] was responsible for its interaction with LAPTM5. **(F)** Left, schematic of full-length or mutant LAPTM5; right, Co-IP assays results revealed that the UIM and PY2 motifs of LAPTM5 were responsible for its interaction with ASK1. **(G)** Co-IP assays demonstrated that LAPTM5 inhibits ASK1 N-terminal dimerization in 293T cells transfected with the indicated plasmids. For **(A–G)**, the results are representative of three independent experiments.

To determine whether ASK1 is a direct target of LAPTM5, Flag-tagged LAPTM5 and HA-tagged ASK1 were overexpressed in HEK293T cells. Our IP experiments demonstrated that LAPTM5 co-immunoprecipitated with ASK1 and vice versa (Figures 8A,B). Furthermore, the GST pull-down assay confirmed a direct interaction between LAPTM5 and ASK1 (Figures 8C,D). Subsequently, molecular mapping assays were conducted to identify the protein domains responsible for LAPTM5 and ASK1 association. First, we generated two truncated HA-tagged ASK1 constructs. Next, Flag-tagged LAPTM5 was co-transfected with these ASK1 deletion mutants followed by co-immunoprecipitation (co-IP). On the one hand, results demonstrated that the N-terminus of ASK1 [ASK1(1-678)] was required for its direct interaction with LAPTM5 (Figure 8E). On the other hand, Laptm5 contains three polyproline–tyrosine (PY) motifs (L/PPxY) and a ubiquitin interacting motif (UIM). As indicated in previous studies, these protein domains are closely associated with LAPTM5 function and cellular location (Pak et al., 2006; Ouchida et al., 2008). Accordingly, we generated five Flag-tagged LAPTM5 mutants and co-transfected them with HA-tagged ASK1 followed by co-IP in HEK293T cells. These mutants include mUIM (Q232A, V235G, and L236A), mPY1 (Y120A), mPY2 (Y239A), mPY3 (Y259A), and mPY1-3 (Y120A, Y239A, and Y259A; Figure 8F). The mapping analysis indicated that both the UIM and PY2 domains were responsible for the interaction of LAPTM5 with ASK1 (Figure 8F). Unexpectedly, the three PY motifs mutants (mPY1-3) of LAPTM5 retained the ability to bind with ASK1. These observations suggested that mPY1-3 may bind to ASK1 through other pathways.

The N-terminal domains of ASK1 are critical for its N-terminal dimerization, which contributes to ASK1 phosphorylation and activation when subjected to extracellular stimuli. Therefore, to determine whether LAPTM5 regulates the ASK1-JNK/p38 signaling pathway by affecting ASK1 dimerization, we transfected myc-tagged LAPTM5, HA-tagged ASK1(1-678), and Flag-tagged ASK1(1-678) in HEK293T cells followed by co-IP. Results revealed that LAPTM5 inhibited ASK1 N-terminal dimerization (Figure 8G). In conclusion, this evidence suggests LAPTM5 regulates the ASK1-JNK/p38 signaling pathway by directly interacting with ASK1 and inhibiting ASK1 N-terminal dimerization.

## Discussion

The efficacy of stroke treatment has been disappointing, owing to an incomplete understanding of the complex pathophysiological processes in response to cerebral I/R injury. In the present study, we observed that LAPTM5 expression levels decreased *in vivo* and *in vitro* in response to I/R damage. Next, LAPTM5 KO mice were enrolled in our research, and these exhibited larger infarct volumes and more severe neurological deficits. Combined experimental verification and RNA-seq data analysis demonstrated that LAPTM5 ablation exacerbated brain I/R damage by promoting inflammation and apoptosis. *In vitro*, primary cortical neurons were cultured and subjected to OGD/R treatment. LAPTM5 knockdown aggravated neuronal inflammation and apoptosis in primary neurons, while LAPTM5 overexpression ameliorated the aforementioned processes. Mechanistically, KEGG pathway enrichment analysis revealed that the MAPK signaling pathway was mainly involved in LAPTM5 deletion-mediated cerebral I/R injury. In addition, western blot indicated that LAPTM5 deficiency contributed to activating the ASK1-JNK/p38 pathway both *in vivo* and *in vitro*, while LAPTM5 overexpression suppressed this axis in primary neurons. Furthermore, LAPTM5 regulated the ASK1-JNK/p38 signaling pathway by directly interacting with ASK1 and inhibiting ASK1 N-terminal dimerization. ASK1 was identified as a novel direct target of LAPTM5 in the brain in our study. Thus, targeting the LAPTM5-ASK1 axis may become a new therapeutic approach with strong potential.

LAPTM5 has been demonstrated to be upstream of various signaling pathways, participating in orchestrating multiple biological processes. As a smurf2 receptor on the lysosomal membrane, LAPTM5 is involved in inhibiting the TGFβ signaling pathway (Colland et al., 2004). In CD40 positive glioblastoma, LAPTM5 expression could suppress tumor growth by inhibiting CD40-mediated activation of NF-κB (Berberich et al., 2020). Furthermore, LAPTM5 could play an important protective role in pathological myocardial hypertrophy through the Rac1-MEK-ERK1/2 pathway (Gao et al., 2021). On the other hand, in some previous studies, LAPTM5 has been shown to negatively regulate cell survival. In HeLa cells, LAPTM5 overexpression is involved in activating mitochondrial-dependent apoptosis pathways (Jun et al., 2017). Inoue et al. demonstrated that LAPTM5-mediated programmed cell death is closely associated with the favorable prognosis of neuroblastomas (Inoue et al., 2009). However, in a bladder cancer study, LAPTM5 deficiency suppressed cell proliferation and activity (Chen et al., 2017). We also demonstrated that LAPTM5 deficiency could inhibit cell viability and exacerbate apoptosis in primary neurons following OGD/R treatment by activating the ASK1-JNK/p38 pathway. In addition, controversial results were observed regarding the regulation of inflammation. LAPTM5 promotes the activation of NF-κB and MAPK signaling pathways, leading to increased release of pro-inflammatory factors in macrophages (Glowacka et al., 2012). However, it negatively regulates the activation of T and B cells and diminishes cytokine production (Ouchida et al., 2008, 2010). Similarly, in the present study, we demonstrated that LAPTM5 could ameliorate neuronal inflammation by blocking the ASK1-JNK/p38 MAPK signaling pathway. These functional differences in the regulation of apoptosis and inflammation may be due to the different cell types or stimuli, the mechanism of which still needs further exploration.

Previous studies determined that LAPTM5 contains 3 PY motifs and 1 UIM motif; these domains are highly related to their subcellular location and function (Pak et al., 2006; Ouchida et al., 2008, 2010). LAPTM5 interacts with Nedd4 through the PY motif [the third PY motif (PY3) appears most important], and then recruits GGA3 to combine with its laptm5-UIM, allowing LAPTM5 translocation from the Golgi to the lysosome (Pak et al., 2006). Mutation of PY2 (mPY2) or PY3 (mPY3) eliminates the ability of LAPTM5 to downregulate T cell antigen receptor (TCR), and mutation of UIM (mUIM) also reduces the ability of LAPTM5 to degrade TCR (Ouchida et al., 2008). Accordingly, to explore the LAPTM5 domains that interact with ASK1, we constructed four mPY, namely mPY1 (Y120A), mPY2 (Y239A), mPY3 (Y259A), mPY1-3 (Y120A, Y239A, and Y259A), and mUIM (Q232A, V235G, and L236A). Through IP mapping, we showed that mPY2 and mUIM failed to bind to ASK1, demonstrating that the PY2 and UIM domains of LAPTM5 are required for its interaction with ASK1. A previous study also demonstrated that the UIM domain in LAPTM5 contains the PY2 motif (Ouchida et al., 2008). Unexpectedly, the three PY motif mutants (mPY1-3) of LAPTM5 retained the ability to bind with ASK1. These results suggested that mPY1-3 may bind to ASK1 through other pathways. Another study also showed that three PY motif mutations might have opposite functions compared with just one PY motif mutation (Ouchida et al., 2008). However, the mechanism of the interaction between the two molecules needs to be further explored.

Apoptosis signal-regulating kinase 1 (ASK1), a 160-kDa serine/threonine-protein kinase, belongs to the MAP3K family (Ichijo et al., 1997). As an upstream molecule, ASK1 can phosphorylate and thereby activate MKK4/MKK7 and MKK3/MKK6, which in turn activates the JNK/p38 pathway (Ichijo et al., 1997; Tobiume et al., 2001). ASK1-MAPK cascades are in response to various intra- and extracellular stressors (such as inflammatory signals, reactive oxygen species (ROS), lipopolysaccharide (LPS), tumor necrosis factor α (TNFα), endoplasmic reticulum (ER) stress, and calcium overload) to induce cell death, inflammation and differentiation (Matsukawa et al., 2004). ASK1-MAPK signaling pathways are also involved in the pathophysiological process of many diseases (Nagai et al., 2007; Cheon and Cho, 2019; Yan et al., 2019). ASK1 KO mice exhibited resistance to cardiomyocyte death and reduced infarct size in myocardial I/R (Watanabe et al., 2005). In the pathogenesis of neurodegenerative diseases, ASK1 seems to play a critical role in response to ER stress (Sekine et al., 2006). Previous studies have revealed that ASK1 plays an important role in cerebral I/R injury. Using RNA interference to knock down ASK1 in the brain can dramatically protect against cerebral I/R injury and decrease neuronal apoptosis in rats (An et al., 2013). TRAF1 promotes neuronal apoptosis after cerebral I/R through the ASK1-JNK pathway (Lu et al., 2013). ASK1 can increase the permeability of the blood-brain barrier and cause brain edema after cerebral ischemia (Song et al., 2015). ASK1 knockdown can reduce astrocyte activation and glial scaring, which is beneficial to neurological function recovery in response to I/R insult (Cheon et al., 2016). Furthermore, ASK1 can affect the inflammatory response after cerebral ischemia by regulating the polarization state of microglia (Cheon et al., 2017). In the present study, LAPTM5 directly interacted with ASK1 to inactivate the ASK1-JNK/p38 axis, thereby achieving cerebral protection after brain I/R.

Under physiological conditions, ASK1 is in an inactive state. Once stimulated, its N-terminal dimerization and subsequent autophosphorylation activate the JNK/p38 pathway (Tobiume et al., 2001; Ogier et al., 2020). Moreover, TOLLIP interacts with ASK1 to increase its N-terminal dimerization, exacerbating hepatic I/R injury by activating the downstream JNK/p38 signaling pathway (Yan et al., 2019). On the contrary, *N*-acetylgalactosaminyltransferase-4 protects against hepatic I/R injury by inhibiting ASK1 N-terminal dimerization and inactivating the ASK1-JNK/p38 axis (Zhou et al., 2021). In brain I/R, ASK1 undergoes phosphorylation and activation through NO-induced dimerization of ASK1, thereby activating the JNK pathway (Liu et al., 2013). In the present study, LAPTM5 directly interacted with ASK1 (1–678 aa) to inhibit its N-terminal dimerization, which in turn decreased the phosphorylation and activation of the ASK1-JNK/p38 pathway, achieving neuroprotection based on ameliorating inflammation and apoptosis.

There are also some limitations in our study. First, considering that neurons are the most vulnerable cell type after cerebral ischemia and its survival may be the key factor affecting the prognosis of stroke, so we just investigated the role of LAPTM5 in neurons *in vitro*. Since we used global KO mice, we cannot rule out the function of LAPTM5 in microglia and astrocytes in cerebral I/R processes. Also, microglia have been demonstrated to express LAPTM5 (Origasa et al., 2001), inflammatory mediators released from these cells might induce neuronal damages (Origasa et al., 2001). We can further solve this problem by generating cell-specific LAPTM5 knockout or overexpressing mice in the future study. Second, previous studies have demonstrated that ASK1 pathway activation mediated cerebral I/R injury, and inhibition of ASK1 ameliorated this damage (Lu et al., 2013; Cheon et al., 2018a,b). However, we did not explore whether the use of ASK1 inhibitors could rescue the exacerbation of I/R damage caused by LAPTM5 deficiency. Hao et al. (2016) demonstrated that intracerebroventricularly injection of NQDI-1, a specific inhibitor of ASK1, attenuated cerebral ischemia injury by inhibiting cell apoptosis. Thus, it would be beneficial to use ASK1 inhibitors for cerebral I/R injury. However, given that ASK1 signaling could activate JNK/p38, then inhibition of ASK1 will subsequently inhibit JNK and p38 as well, which may cause some side effects. Furthermore, ASK1 inhibition could potentially suppress innate immunity and the role of ASK1 is not fully understood (Tesch et al., 2016), which may place limitations on the therapeutic use of ASK1 inhibitors. Third, we did not elucidate what decreased expression of LAPTM5 under ischemic condition. In this study, we found that both RNA and protein expression levels of LAPTM5 were down-regulated after cerebral I/R injury in mice, indicating that the regulation of LAPTM5 expression may occur at the DNA level. It has been reported that DNA methylation could regulate the expression of LAPTM5 (Hayami et al., 2003; Inoue et al., 2009). In addition, recent studies have found that epigenetics, especially DNA methylation, affects the prognosis of ischemic stroke (Tang and Zhuang, 2019; Stanzione et al., 2020). Endres et al. (2000) found that DNA methylation levels were upregulated after focal cerebral ischemia and correlated with poor prognosis. Pharmacological inhibition or gene knockout of DNA methyltransferases (DNMTs) can alleviate ischemic brain injury and increase neuronal survival (Dock et al., 2015; Sharifulina et al., 2021). In our study, cerebral I/R treatment may increase DNA methylation, resulting in decreased LAPTM5 expression. Of course, the specific mechanism in these processes needs to be further studied. Finally, how can we overexpress LAPTM5 therapeutically in a short time after the start of reperfusion? As far as we know, there two approaches might be able to use for a stroke treatment to increase the expression of LAPTM5. First, generating small activating RNA (saRNA) targeting LAPTM5, which is a chemically synthesized small double-stranded RNA (dsRNA) oligonucleotide of 21 nt in length that positively and reversibly upregulates its target genes beyond endogenous levels (Li et al., 2006; Janowski et al., 2007; Tan et al., 2021). saRNA are small, versatile and safe, they represent a new class of therapeutics that can rescue the downregulation of critical genes in disease settings. saRNA for LAPTM5 could be injected right after a stroke happened to prevent the reperfusion damage. Second, synthesizing LAPTM5 protein *in vitro*, and delivery this protein into the brain predicted ischemic penumbra by intracranial injection after the start of reperfusion. Using BDNF protein synthesized *in vitro* and injecting into the brain has been approved useful to rescue the impairment of extinction memory in mice (Li et al., 2019). Therefore, LAPTM5 protein direct injection could be another possible way to protect the brain from reperfusion damage.

## Conclusion

We demonstrated that LAPTM5 might serve a neuroprotective role during cerebral I/R injury. Furthermore, the mechanism by which LAPTM5 regulates brain I/R may depend on the ASK1-JNK/p38 signaling pathway, making LAPTM5 a highly promising method for ischemic stroke treatment.

## Data availability statement

The datasets presented in this study can be found in online repositories. The names of the repository/repositories and accession number(s) can be found below: https://www.ncbi.nlm.nih.gov/genbank/, PRJNA811709.

## Ethics statement

The animal study was reviewed and approved by Animal Care and Use Committee of Zhongnan Hospital of Wuhan University.

## Author contributions

ZZ, WZ, and JC designed the experiments. ZZ, LW, ZW, YZ, and CX performed the experiments. ZZ, LW, ZW, MS, and TZ analyzed the data. ZZ and WZ wrote the original draft. JC, XL, and WW edited and reviewed the final manuscript. All authors read and approved the final manuscript.

## Abbreviations

ASK1: apoptosis signal-regulating kinase 1
Bad: BCL2-associated agonist of cell death
Bax: BCL-2-associated X protein
Bcl2: B-cell lymphoma-2
CCK-8: cell counting kit-8
Ccl-2: C-C motif chemokine ligand 2
Ccl-5: C-C motif chemokine ligand 5
IP: immunoprecipitation
I/R: ischemia-reperfusion
ERK: extracellular signal-regulated kinase
Fas: TNF receptor superfamily member 6
IKK β: inhibitor of nuclear factor-kappa B kinase beta
I κ B α: inhibitor of nuclear factor-kappa-B α
Il6: Interleukin 6
JNK: c-JunN-terminal kinase
LAPTM5: lysosomal-associated protein transmembrane 5
MAPK: mitogen-activated protein kinase
NF- κ B: nuclear factor kappa B
OGD/R: oxygen and glucose deprivation/reoxygenation
p38: p38 mitogen-activated protein kinase
p65: nuclear factor kappa-B RelA
TAK1: transforming growth factor- β-activated kinase 1
Tnf: tumor necrosis factor
tMCAO: transient middle cerebral artery occlusion
TTC: 2,3,5-triphenyl-2H-tetrazolium chloride
TUNEL: terminal deoxynucleotidyl transferase dUTP nick end labeling.
